# Multifaceted implementation strategy to improve the evaluation of penicillin allergies in perioperative patients: a pre-post feasibility implementation study

**DOI:** 10.1017/ice.2024.119

**Published:** 2024-12

**Authors:** Eileen J. Carter, Katherine Zavez, Carol Schramm, Meagan M. Zolla, Katelyn Baron, David B. Banach

**Affiliations:** 1 University of Connecticut School of Nursing, Storrs, CT, USA; 2 University of Connecticut Department of Statistics, Storrs, CT, USA; 3 UConn John Dempsey Hospital, Farmington, CT, USA; 4 University of Connecticut School of Medicine, Farmington, CT, USA; 5 Yale School of Public Health, New Haven, CT, USA

## Abstract

**Objective:**

The U.S. Centers for Disease Control and Prevention encourages nurses to evaluate penicillin allergies as part of hospital-based antibiotic stewardship programs. We evaluated the feasibility of an implementation strategy to improve nurses’ comprehensive documentation of penicillin allergies. We defined feasibility as the uptake and acceptability of documentation procedures.

**Design:**

Six-month pre-post feasibility implementation study.

**Setting:**

Outpatient surgical areas of an academic medical center located in the U.S.

**Intervention:**

The implementation strategy was guided by the Capability, Opportunity, Motivation Model for Behavior Change and included, building an interdisciplinary coalition to iteratively evaluate the implementation effort, educational meetings with surgical prescribers and perioperative nurses, the development and distribution of educational pocket cards, and structured communication messages in the electronic medical record.

**Results:**

A total of 426 patients with 487 penicillin allergy records (216 records pre-implementation period, 271 records post-implementation period) were analyzed. Penicillin allergy documentation contained the following information in the pre- versus post-implementation period: symptoms of the reaction (87% vs 87%), timing/years since reaction (8% vs 26%), onset of reaction in relation to taking penicillin (0% vs 21%), how symptoms resolved (0% vs 21%), and penicillin re-exposure (3% vs 21%). Focus groups revealed nurses perceived documentation procedures as highly acceptable. Major drivers of acceptability included the perceived effectiveness of a detailed allergy history and self-efficacy in conducting a detailed allergy history.

**Conclusions:**

Nurses perceived the comprehensive documentation of penicillin allergy history intervention as acceptable, and uptake improved following a theory-informed implementation strategy. We offer implementation strategy components to facilitate nurses’ engagement in penicillin allergy evaluation.

## Introduction

Nearly 32 million adults in the U.S. report an allergy to penicillin and formal penicillin allergy testing reveals only 5% of patients who report a penicillin allergy have a true allergy to penicillin.^1,2^ Patients reporting a penicillin allergy receive less effective and more costly antibiotic treatment, and are at increased risk for the development of *Clostridioides difficile* infection and infections with antibiotic-resistant bacteria.^3,4^ Penicillin allergies are particularly consequential among patients undergoing surgery. Surgical patients with a documented penicillin allergy versus those without a documented penicillin allergy are more likely to receive less-effective, second-line, prophylactic antibiotic treatments that increase their risk for mortality, surgical site infections, and longer hospital stays.^5,6^ Comprehensive penicillin allergy histories enable the risk-stratification and appropriate management of reported penicillin allergies.^2^ Yet, up to 40% of documented penicillin allergy histories lack descriptions of the index reaction,^7^ which impedes risk stratification and perpetuates the unnecessary avoidance of penicillins and cephalosporins.

In 2019, the U.S. Centers for Disease Control and Prevention (CDC) Core Elements for Hospital Antimicrobial Stewardship Programs specified that nurses may play an important role in stewardship by improving the evaluation of reported penicillin allergies.^8^ We developed a multifaceted implementation strategy (actions to facilitate the adoption of evidence-based interventions) to amplify facilitators and minimize barriers to nurses’ evaluation of reported penicillin allergies.^9–12^ Our development of the implementation strategy was guided by the Capability, Opportunity, Motivation Model for Behavior Change (COM-B) model of behavior change that specifies behavior results from three interacting components,^13^ ie, Capability (nurses’ knowledge and skill to evaluate penicillin allergies), Opportunity (external factors that influence nurses’ ability to evaluate penicillin allergies), and Motivation (nurses’ emotional responses and analytic decisions to evaluating penicillin allergies). We defined nurses’ evaluation of reported penicillin allergies as nurses’ implementation of two interventions: 1) documenting penicillin allergies using the STORY mnemonic (Symptoms of allergy, Timing of allergy, Onset of symptoms, Resolution, and Yet again use of penicillin)^14^ and 2) notifying prescribers of patients with low-risk symptoms of reported penicillin allergy.^2^ We provide a rationale for and description for interventions in Supplemental Materials, Appendix A.

The implementation strategy included five components and targeted nurses’ capability, opportunity, and motivation to comprehensively evaluate penicillin allergies. We categorize implementation strategy components according to the Expert Recommendations for Implementing Change (ERIC) project,^15^map implementation strategy components to COM-B, and describe implementation strategies in Table 1. First, we built an interdisciplinary coalition to purposely develop, examine, reexamine, and adjust implementation strategy components. Second, we hosted educational meetings with surgeons, perioperative advanced practice nurses, perioperative physician assistants, anesthesiology physicians, nurse anesthetists, clinical pharmacists, and perioperative nurses. Educational meetings with surgical prescribers oriented them to and ensured their support of nursing interventions. Educational meetings with perioperative nurses covered the following topics: prevalence and harms of misclassified penicillin allergies, recommendations that nurses improve the evaluation of reported penicillin allergies, the STORY mnemonic to improve penicillin allergy histories, and an evidence-based toolkit that specifies low-risk symptoms of penicillin allergy, See Supplemental Materials, Appendix B.^2,8,14^ Third, we developed dot phrases in the electronic medical record to minimize documentation burden associated with the interventions. Fourth, we made and distributed educational pocket-cards for nurses that outlined the STORY mnemonic, symptoms of low-risk penicillin allergies, and associated dot phrases. Lastly, we provided monthly feedback to nurses to further encourage intervention uptake. We provided a rationale for implementation strategy components in Supplemental Materials, Appendix C. In this study, we aimed to evaluate the feasibility of the implementation strategy as defined by perioperative nurses’ uptake of interventions and the acceptability of interventions as experienced by key stakeholders.

**Table 1.** Implementation strategy components, mapping to COM-B, and implementation strategy description

Implementation strategy component	Mapping to COM-B^*^			Implementation Strategy Description
	C	O	M	
Build a coalition	X	X	X	• Team members engaged in bi-weekly 45-minute videoconference meetings over the course of the study period to contribute to the inception, execution, reexamination, and refinement of the implementation effort.
Purposely reexamine the implementation	X	X	X	• Team members included an infectious disease physician, nurse educator, professional nurse, nurse leader, and nurse researcher.
Inform local opinion leaders			X	• Team members informed hospital nursing leadership and the antimicrobial stewardship committee of the project during the planning and implementation period via videoconference and/or email.
				• We conducted formative evaluations with members of the antimicrobial stewardship committee during the implementation period to solicit their feedback with the initiative.
Conducted educational meetings with perioperative nurses and surgical providers	X		X	• A one-time, 30-minute educational meeting was hosted with perioperative nurses and began the formal implementation period. Topics covered: the prevalence and harms of misclassified penicillin allergies, importance of comprehensive penicillin allergy assessments, CDC recommendations for nurses to improve the evaluation of reported penicillin allergies, examples of penicillin antibiotics, the STORY mnemonic to improve penicillin allergy histories, and low-risk symptoms of reported penicillin allergy.
				• A one-time, 10-minute, videoconference educational meeting was hosted with surgical providers to orient them to the initiative encouraging nurses’ evaluation of penicillin allergies.
				• Educational meetings were led by EC and DB.
Developed and distributed educational materials (ie, pocket cards)	X	X		• Perioperative nurses received pocket cards during the educational meeting.
				• Pocket cards included the STORY mnemonic detailing penicillin allergy assessment fields, low-risk symptoms of penicillin allergy, and dot phrases in EPIC (electronic health record used in the study facility) to facilitate nurses’ documentation of interventions.
Change record systems	X	X		• Dot phrases in EPIC were announced during the educational meeting with perioperative nurses to facilitate nurses’ enactment of interventions.
				• Dot phrases included the elements of STORY and a structured communication message for nurses to send to providers regarding patients with low-risk symptoms of reported penicillin allergy.
Obtain and use feedback			X	• Approximately each month during the post-implementation period, perioperative nurses were provided with positive feedback concerning nurses’ implementation of practices.
				• Feedback was gathered by the study team and included informal feedback they received from key opinion leaders. Positive feedback included provider comments regarding the likelihood of STORY to improve patient care, feedback from pharmacists concerning their use of STORY in clinical care, and feedback from nursing leaders regarding the importance of perioperative nurses’ involvement in the initiative.

* COM-B refers to the Capability, Opportunity, and Motivation Model of Behavior.

## Methods

This was a single-site, pre-post, feasibility implementation study conducted in the outpatient surgical areas of an academic medical center the Northeast United States. At the time of the study period, penicillin allergies were documented in the allergy module of the electronic medical record (EPIC) and there was a lack of formal partnership between the antimicrobial stewardship program and the department of nursing in addressing antibiotic allergy assessment and documentation. We used the Standards for Reporting Implementation Studies (StaRI) to guide the reporting of our implementation study. See Supplemental Materials, Appendix D.

The pre-implementation period was from October 28, 2022, to January 24, 2023, and the post-implementation period was from January 25, 2023, to April 23, 2023. We retrospectively obtained the structured and unstructured penicillin allergy documentation of eligible outpatient surgical patients at the study site during the study period. Patients were eligible for study inclusion if they had a documented allergy to penicillin, amoxicillin, ampicillin, nafcillin, oxacillin, or piperacillin in the electronic medical record, presented from an outpatient setting, and underwent a surgical procedure during the study period. We excluded duplicate records, patients with cancelled outpatient procedures, and patients captured in the pre-and post-implementation periods (as details of penicillin allergy record updates were unspecified in our data pull).

Two researchers (EC and KZ) independently characterized the information contained in each penicillin allergy record according to the STORY mnemonic.^14^ We then used percentage agreement to determine the level of agreement between researchers by dividing the number of records in agreement by the total number of records and multiplying by 100. Using published toolkits,^2^ we identified patients that met low-risk penicillin allergy criteria in the post-implementation period. Low-risk penicillin allergies included the following symptoms and/or descriptions: nausea, vomiting, diarrhea, headache, fatigue, itchiness, patient has no recollection of allergy, family history of allergy, and yeast infections.^2^ Records containing no symptoms (ie, field was blank or patient was unable to specify symptoms) were classified as having uninterpretable risk. Among patients with low-risk penicillin allergies, we evaluated nurses’ use of a structured note, which documented their notification of prescribers concerning the low-risk allergy. Our primary outcome was nurses’ comprehensive documentation of penicillin allergy histories, in which we compared the information contained in penicillin allergy documentation pre- and post-implementation period. We used a 3-month pre, post-test design to achieve the recommended sample size for feasibility studies to estimate group differences and allow key stakeholders sufficient exposure to implementation strategies.^16^ We also quantified nurses’ notification of prescribers concerning low-risk penicillin allergies by determining the proportion of patients meeting low-risk penicillin allergy criteria for whom nurses notified prescribers.

We conducted informal formative evaluations with key stakeholders as recommended in implementation studies to learn the experiences of those directly impacted by the implementation effort and to judge the need for refinements to the implementation strategy.^17^ During the implementation period, we attended a regularly scheduled perioperative nurse meeting and a regularly scheduled antimicrobial stewardship committee meeting, in which team members provided a brief overview of the status of the initiative and asked attendees to share their experiences and feedback concerning the initiative. Formative evaluations were attended by a minimum of two study team members to ensure a shared understanding of conversation and to reexamine the implementation effort with the larger team.

**Figure 1.**
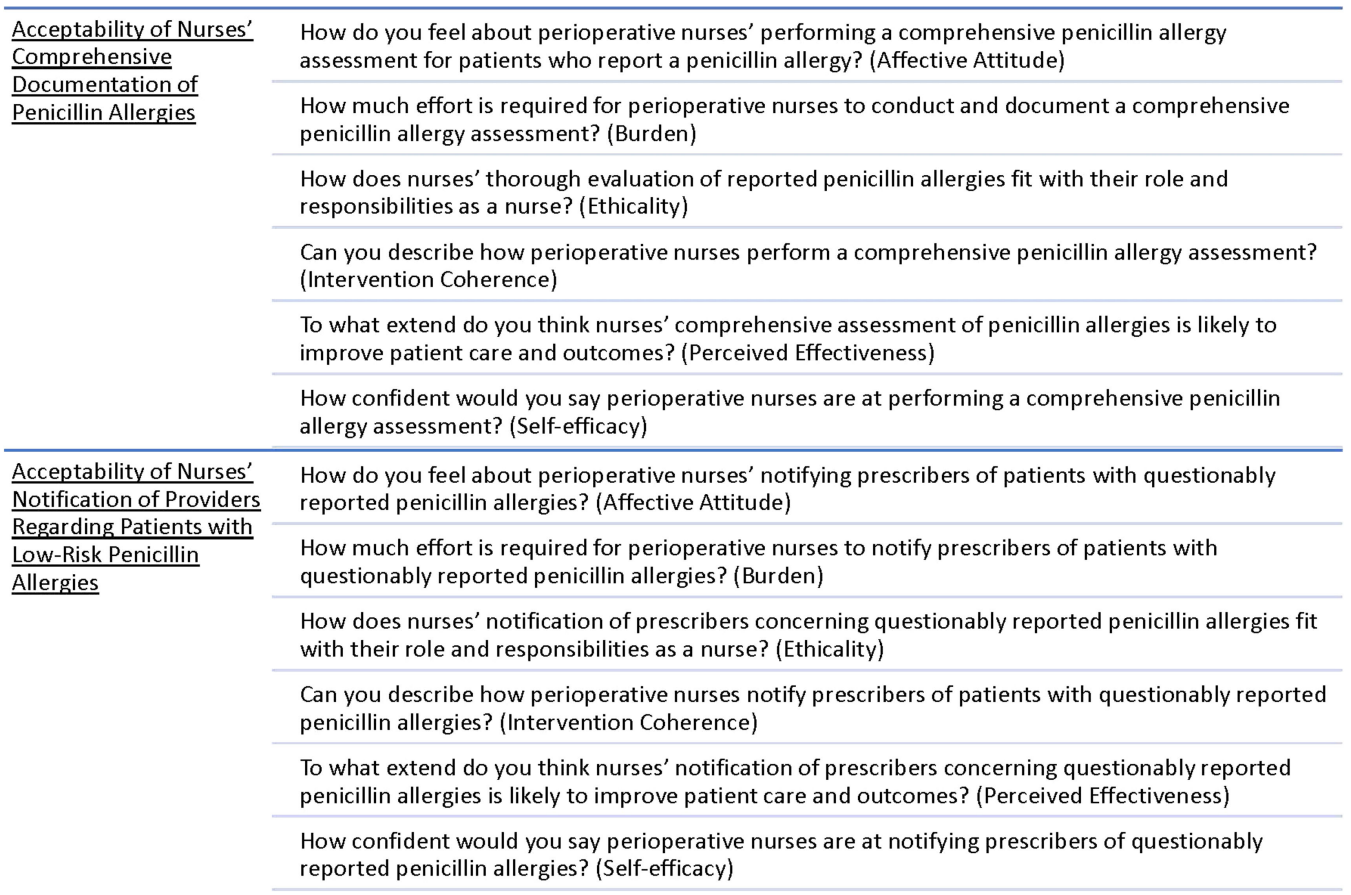
Focus group guide.

At the end of the study period, we conducted one focus group with perioperative nurses (N = 7) to understand the acceptability of interventions. An experienced qualitative researcher (EC) facilitated focus group discussion and a second researcher (KF) maintained field notes of contextual information and insights gained during focus group discussion.^18^ Focus group questions were informed by the Theoretical Framework of Acceptability^19^ and addressed constructs of intervention acceptability, ie, affective attitude, burden, ethicality, intervention coherence, perceived effectiveness, and self-efficacy. See Focus Group Guide, Figure 1. To enhance the credibility of findings, we conducted member checking during the focus group,^20^ in which EC summarized in her own words participants’ descriptions of intervention acceptability and asked participants to comment on the accuracy of the summary. Focus groups were audio recorded, transcribed by a professional transcription service and analyzed using thematic analysis according to the Theoretical Framework of Acceptability.^21^ The academic medical center’s institutional review board approved this study (IRB # 23-027S-1).

## Results

The implementation strategy reached a total of 171 nurses and prescribers from the operating room and anesthesia (n = 49), obstetrics (n = 30), pharmacy (n = 23), antimicrobial stewardship (n = 22), orthopedics (n = 20), pre-op (n = 8), otolaryngology (n = 7), general surgery (n = 6), and vascular surgery (n = 6). A total of 426 patients with 487 penicillin allergy records (216 records pre-implementation period, 271 records post-implementation period) met eligibility criteria and were analyzed. The drug implicated in allergy records were listed as a specific penicillin drug (eg, penicillin V, penicillin G, etc.,) or “penicillins” (n = 355, 73%), amoxicillin or an amoxicillin combination drug (n = 126, 26%), and ampicillin (n = 6, 1%). Among records, 424 (87%) contained a description of the penicillin allergy reaction. The most common symptoms documented were: rash (n = 150, 31%), hives (n = 131, 27%), gastrointestinal symptoms (n = 71, 15%), anaphylaxis (n = 46, 9%), swelling (n = 37, 8%), and itching (n = 34, 7%).

Penicillin allergy records contained the following information in the pre- versus post-implementation period: symptoms of the reaction (87.0% vs 87.1%), timing/years since reaction (7.9% vs 25.8%), onset of reaction in relation to taking penicillin (0% vs 20.7%), how symptoms resolved (0% vs 20.7%), and penicillin re-exposure (2.8% vs 21.0%), Table 2.

**Table 2.** Content of penicillin allergy records pre versus post implementation strategy

Information Contained in Penicillin Allergy Records*	Penicillin Allergy Records Pre-Implementation n = 216		Penicillin Allergy Records Post Implementation n = 271		Difference in Documentation from Post to Pre-Implementation Period
	n	%	n	%	Post - Pre, (95% CI)
S - Symptoms of reaction	188	87.04	236	87.08	0.04 (–6.00 – 6.10)
T - Timing (years) since reaction	17	7.87	70	25.83	17.96 (11.22 – 24.70)
O - Onset of reaction after penicillin	0	0	57	21.03	21.03 (15.77 – 26.30)
R - Resolution of symptoms	0	0	56	20.66	20.66 (15–.43 – 25.90)
Y - Yet again (re-exposure to penicillin)	6	2.78	57	21.03	18.26 (12.52 – 24.00)

* STORY mnemonic used to characterize the information contained in penicillin allergy records.

Percentage agreement in evaluating the content of penicillin allergy documentation ranged from 97% to 100% across STORY fields.

In the post-implementation period, 53 patients (20%) met low-risk penicillin allergy criteria. Reasons for low-risk criteria included, gastrointestinal symptoms (n = 37, 70%), itchiness (n = 9, 17%), yeast infections (n = 5, 9%), and patient denying allergy (n = 2, 4%). Nurses documented their notification of prescribers for 14 (26%) of patients meeting low-risk penicillin allergy criteria. Symptoms of allergy were entered as structured data in the pre- and post-implementation period (n = 174, 80.6% records pre-implementation; n = 216, 79.7% records post-implementation). Free-text information was provided in 69 (31.9%) of records in the pre-implementation period and 121 records (44.7%) in the post-implementation period, of which 56 records (46.2%) included the use of the.STORY phrase.

Formative evaluations with members of the hospital antimicrobial stewardship committee and perioperative nurses revealed their positive experiences with the initiative. One pharmacist noted caring for a patient with STORY information and described it as “*So helpful*.” Nurses described using STORY in practice, pointed to the STORY pocket cards that appeared on their ID badge clips, and reflected that the STORY pocketcard was taped to nurse workstations. Perioperative nurses also recommended that a listing of penicillin-type antibiotics be posted to workstations to remind nurses of the specific antibiotic allergies that are targeted for STORY. This was the only modification made to the implementation strategy.

In the focus group, nurses described their comprehensive assessment of penicillin allergy histories as highly acceptable. We provide the drivers of intervention acceptability and representative quotes in Table 3. Major drivers of intervention acceptability included the appropriate ethicality of the intervention, high self-efficacy to perform the intervention, and the perceived effectiveness of the intervention. Nurses reported that taking a detailed penicillin allergy history fit well with the nurses’ role of communicating important patient information to prescribers to guide patient care. Nurses also expressed confidence in their ability to use STORY to gather a detailed penicillin allergy history, although noted that time constraints and poor patient recall presented barriers to STORY detail and completion. In describing poor patient recall as a barrier, one nurse said, “*There’s a lot of [patients] who had it when they were little and they can’t even remember it.”* Despite these barriers, nurses believed their thorough documentation of penicillin allergies would improve patient care in the future as prescribers could use the information for antibiotic selection. Nurses cited the STORY mnemonic, pocket card, and EPIC dot phrase as helpful in recording a detailed penicillin allergy history.

**Table 3.** Drivers of intervention acceptability and representative quotes

*Intervention: Nurses’ Taking a Detailed Penicillin Allergy History*	
**Positive affective attitudes** - Nurses expressed favorable experiences and attitudes toward taking a detailed penicillin allergy history.	*“Whenever I see penicillin allergy, I ask the patient…the whole story.” - Respondent 7*
	*“I think that this information’s great information and I think it’s pertinent.” - Respondent 1*
**Positive ethicality** - Nurses’ taking a detailed penicillin allergy history was perceived to fit well with the nurses’ role of communicating important patient information to prescribers.	*“I think it’s important for us to document details, what really happened when they take it so the provider can read it.” - Respondent 3*
**High perceived effectiveness** - Nurses believed comprehensive penicillin allergy histories were likely to improve patient care in the future.	*“The provider will be able to look at it and say, okay, I can [prescribe penicillin] it was just a GI issue. Down the line they would be looking at that more than first thing [before surgery] when they’ve got a thousand things on their mind.” - Respondent 6*
**High self-efficacy** - Nurses expressed a high degree of confidence in their ability to complete STORY.	*“It’s pretty simple. Just follow it step by step by step.” - Respondent 2*
*Intervention: Nurses’ Notification of Prescribers Regarding Low-Risk Penicillin Allergies*	
**Negative affective attitudes** - Nurses described indifferent or negative attitudes when describing their notification of prescribers regarding low-risk penicillin allergies.	*“By the time it gets here it’s kind of like orders are in. You know what I mean?… You just have to start earlier.” - Respondent 4*
	*“[Providers] just like give you attitude like, “We know. Why bother telling us?” They discourage us to go forward. - Respondent 3*
**Poor ethicality** - The determination of low-risk allergy status was perceived as a prescribing clinician—not a nursing responsibility.	*“Providers are the one who make a decision whether it’s low risk or not instead of the nurse making a decision whether it’s low risk or high risk.” - Respondent 3*
**Perceived ineffectiveness of intervention** - Nurses described the need to better engage prescribers and patients to effectively manage low-risk penicillin allergies.	*Lack of prescriber engagement - “There’s no change in the plan. That’s the thing… we tell them, but it doesn’t change the fact that there’s a penicillin allergy on their chart and that they’re not going to order that. - Respondent 1*
	*Lack of patient engagement - “They want it kept on cause whatever the side effect was, they don’t want it again.” - Respondent 5*
**Low self-efficacy** - Nurses expressed a lack of confidence in identifying patients with low-risk penicillin allergies.	*“I don’t feel confident saying…the patient’s low risk because you never know…I feel uncomfortable to tell them it’s low risk. What if something happens…?” - Respondent 7*

Nurses’ notification of prescribers regarding low-risk penicillin allergies had poor acceptability. Drivers of poor intervention acceptability included low self-efficacy to perform the intervention, the perception that the intervention did not fit with nursing responsibilities and roles (poor ethicality), and the perception that the intervention was ineffective. Nurses expressed a lack of confidence in identifying patients with low-risk penicillin allergies and perceived the risk-stratification of penicillin allergies to be a prescribing clinician—not nursing responsibility. Similarly, nurses perceived their notification of prescribers to have no impact on patient care as antibiotic prophylaxis orders remained unchanged and as penicillin allergy records persisted without update. To improve the management of low-risk penicillin allergies, nurses highlighted the need to better engage prescribers and patients in recategorizing allergies as appropriate (side effect vs allergy). Drivers of intervention acceptability and representative quotes are provided in Table 3. We are aware of no harms or unintended effects resulting from the study.

## Discussion

To the best of our knowledge, this is the first study to evaluate the feasibility of an implementation strategy to support recommendations posed by the CDC encouraging nurses to comprehensively evaluate penicillin allergies.^8^ We found perioperative nurses’ documentation of penicillin allergy histories using the STORY mnemonic was acceptable and resulted in improvements in penicillin allergy documentation, whereas perioperative nurses’ notification of prescribers regarding patients with low-risk penicillin allergy symptoms had poor acceptability, despite nurses’ notifying prescribers of patients with low-risk allergies. Defining intervention elements and evaluating their acceptability and tolerability are foundational to progressing from proof-of-concept studies to effectiveness trials.^22^ To date, efforts to improve nurses’ evaluation of penicillin allergies have examined nurses’ use of a penicillin allergy risk-stratification algorithm, nurses’ initiation of a penicillin allergy delabeling questionnaire, and nurses’ monitoring of patients undergoing penicillin allergy testing, with mixed results.^23–25^ To better understand nursing practice(s) that have the greatest promise for antimicrobial stewardship, we analyzed the acceptability and implementation of two nursing interventions to improve the evaluation of penicillin allergies. We found nurses’ evaluation of penicillin allergies using the STORY mnemonic was highly acceptable and our implementation strategy provides the nuts and bolts for how to engage nurses in this practice, thus addressing calls to improve the evaluation and documentation of penicillin allergies.^8,26–29^

Drivers of intervention acceptability among nurses included self-efficacy and the perceived effectiveness and ethicality of interventions. Nurses believed their thorough documentation of penicillin allergies would improve patient care and cited the STORY mnemonic and associated dot phrase as “*straightforward*” and “*easy to follow*”. Recent studies show the importance of STORY fields in predicting penicillin allergy status. A machine learning model using retrospective data from the U.S. found its ability to predict positive penicillin allergy skin testing was strongest when model variables included the symptoms of reaction (particularly hives/urticaria), sex (female), time since reaction, and treatment received for reaction.^30^ Similarly, a model developed and validated using retrospective and prospective data from Australia and the U.S. found time since reaction, symptoms of reaction (anaphylaxis, angioedema, severe cutaneous adverse reaction), and treatment received for reaction had a negative predictive value of 96.3.^31^ Lastly, a multivariate logistic regression model using retrospective data from the UK found the absence of anaphylaxis, time since reaction, and unknown name of the index drug had a negative predictive value of 98.4%.^32^ Future research may use natural language processing and STORY information to further improve the accuracy of these models.

Nurses’ notification of prescribers concerning patients with low-risk penicillin allergies had modest uptake (nurses notified prescribers of 14/53 patients identified as low-risk in post-implementation period), which likely reflects nurses’ descriptions of low self-efficacy to perform the intervention, poor ethicality of the intervention, and the perception that the intervention was ineffective. While the implementation strategy included education on low-risk penicillin allergy criteria that were agreed upon by boards of the American Academy of Allergy, Asthma, and Immunology (AAAAI), the Infectious Diseases Society of America (IDSA), and the Society for Healthcare Epidemiology of America (SHEA),^2,14^ nurses described hesitancy in classifying penicillin allergies as low-risk. Nurses also perceived their implementation of the intervention to have no effect on surgical prescriber behavior, which reflects limitations of the implementation strategy. Surgical prescribers were made aware of nursing interventions but were not given specific guidance on the evidence-based management of low-risk penicillin allergies. It is possible that the acceptability of this intervention would have been greater had we more purposefully engaged prescribers.

As a feasibly study, we identified aspects of the implementation strategy that require modification, namely more potent interventions that target nurse self-efficacy and the perceived effectiveness of nurses’ notification of prescribers regarding low-risk penicillin allergies. More frequent and structured formative evaluations may foster an earlier awareness of aspects of the implementation strategy that are working and those in need of refinement.^17^ Future studies are needed to determine the effectiveness of nurses’ evaluation of penicillin allergies on clinical outcomes among patients, while identifying optimal implementation strategy approaches. Such effectiveness-implementation hybrid designs allow for the dual evaluation of intervention effectiveness and implementation outcomes^33^ and have been used in studies to implement prevention bundles for non-ventilator-associated hospital-acquired pneumonia and screening strategies for sexually transmitted infections.^34,35^

### Study strengths and limitations

This study has several strengths. Our implementation strategy was guided by the COM-B model of behavior change and our evaluation of intervention acceptability was guided by the Theoretical Framework of Acceptability,^13,19^ which provided a comprehensive structure to systematize research procedures and guide further inquiry. Similarly, we characterized implementation strategies using definitions posed by the ERIC project to facilitate the replication and comparison of these strategies in further study. While we did not conduct a formal economical evaluation of the implementation strategy, costs were limited to the printing of educational pocket cards and resources associated with educational training. The minimal costs associated with the initiative support future scalability. Because this study was limited to one setting, the external generalizability of results is unknown. Similarly, because of practical considerations, we conducted informal formative evaluations with key stakeholders and only one formal summative evaluation (focus group) with perioperative nurses. While our process evaluation did not lend itself to data saturation,^36^ similar perspectives and experiences were conveyed from participants and member checking supported the credibility of study findings. It is also possible our data pull of patients with a penicillin allergy missed relevant records. Although we used the same EPIC data query pre- and post-implementation, thereby reducing systematic error.

## Conclusion

In this feasibility study, we found nurses’ thorough documentation of penicillin allergies was highly acceptable and improved following a theory-informed implementation strategy. We offer an innovative, theory-informed approach to improve penicillin allergy documentation and include implementation strategy components to foster adoption among those interested in engaging nurses to improve the evaluation of penicillin allergies.

## Supporting information

Carter et al. supplementary material Carter et al. supplementary material

## Supplementary material

The supplementary material for this article can be found at https://doi.org/10.1017/ice.2024.119.
