# Supplementary material for: Multifaceted implementation strategy to improve the evaluation of penicillin allergies in perioperative patients: a pre-post feasibility implementation study
Source: Infect Control Hosp Epidemiol. 2024 Oct 30;45(12):1455–61. doi: 10.1017/ice.2024.119 (PMC11663472; doi:10.1017/ice.2024.119)
Supplement: Carter et al. supplementary material [file S0899823X24001193sup001.pdf]

Appendix A. Intervention Descriptions and Rationale

Intervention #1: Nurses document penicillin allergies using the STORY mnemonic.

Rationale: Nurses' documentation of penicillin allergies using the STORY mnemonic was targeted for implementation because the STORY mnemonic enables the risk-stratification of penicillin allergies, the mnemonic was perceived as useful by nurses and prescribers in preliminary work, and nurses have expressed a willingness to improve the comprehensive documentation of penicillin allergies.[1-3]

Description: When a nurse cared for a patient who reported a penicillin allergy, the nurse would document the penicillin allergy history using the STORY mnemonic as free-text in the electronic medical record, **Figure 1**.

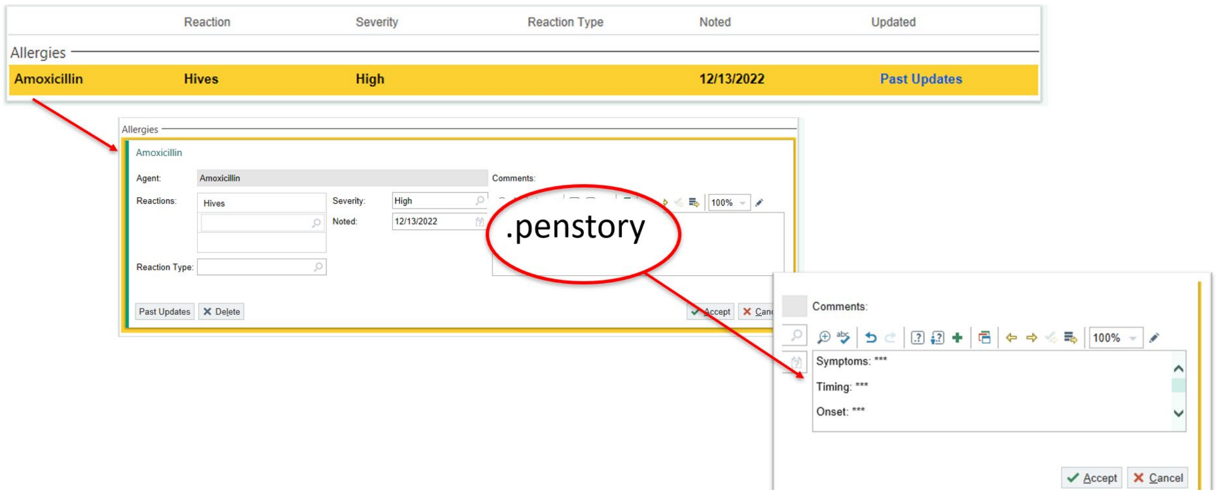

Figure 1. Nurses' Documentation of Penicillin Allergies Using STORY

Intervention #2: Nurses notify prescribers of patients with low-risk symptoms of reported penicillin allergy.

Rationale: Nurses' notification of prescribers of patients with low-risk symptoms of reported penicillin allergy was targeted for implementation because nurses routinely evaluate and review their patient's allergy histories and it's been recommended that nurses notify providers of penicillin allergy labels that may be inaccurate.[4,5]

Description: When a nurse cared for a patient who reported a penicillin allergy and had low-risk symptoms (e.g., isolated gastrointestinal symptoms, headache, fatigue, itchiness, family history of penicillin allergy, patient has no recollection of allergy despite it being in the chart),[1] nurses would verbally alert provider that the patient had a low-risk symptom of penicillin allergy and create an Antimicrobial Stewardship note documenting the low-risk allergy, Figure 2.

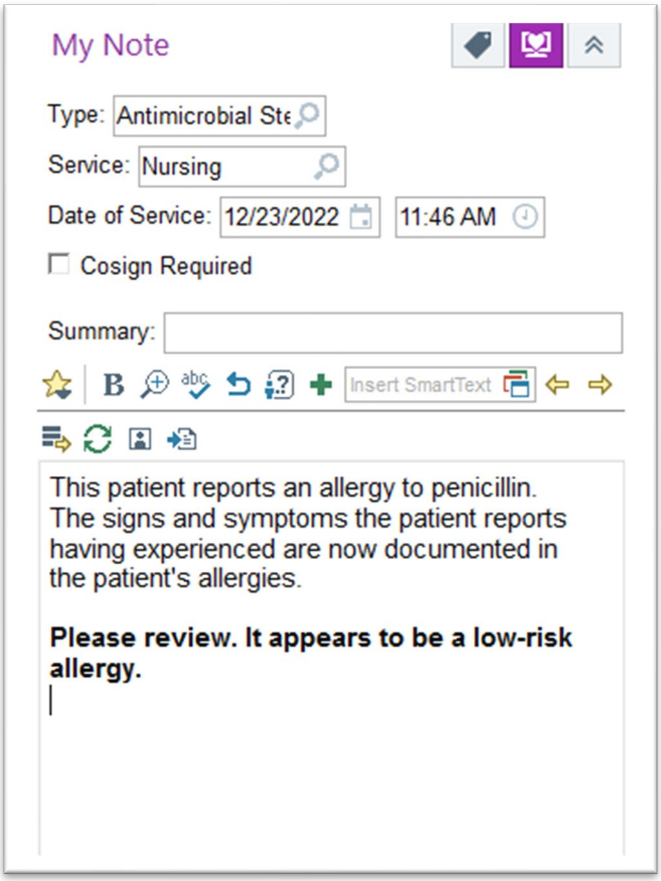

Figure 2. Nurses' Use of an Antimicrobial Stewardship Note to Document Low-Risk Penicillin Allergy

1. Shenoy ES, Macy E, Rowe T, Blumenthal KG. Evaluation and Management of Penicillin Allergy: A Review. *Jama*. 2019;321(2):188-199.
2. Carter EJ, Schramm C, Baron K, Zolla MM, Zavez K, Banach DB. Perceived usefulness of a mnemonic to improve nurses' evaluation of reported penicillin allergies. *Antimicrob Steward Healthc Epidemiol*. 2023;3(1):e124.

3. Carter EJ, Greendyke WG, Furuya EY, et al. Exploring the nurses' role in antibiotic stewardship: A multisite qualitative study of nurses and infection preventionists. *American journal of infection control*. 2018;46(5):492-497.
4. Monsees E, Popejoy L, Jackson MA, Lee B, Goldman J. Integrating staff nurses in antibiotic stewardship: Opportunities and barriers. *American journal of infection control*. 2018;46(7):737-742.
5. Olans RN, Olans RD, DeMaria A, Jr. The Critical Role of the Staff Nurse in Antimicrobial Stewardship—Unrecognized, but Already There. *Clinical Infectious Diseases*. 2015;62(1):84-89.

Appendix B. Nurse Educational Training

Improving the Evaluation of Reported Penicillin Allergies

David Banach, MD
Eileen Carter, PhD, RN

1

Reported allergies to penicillin are common, mostly untrue, and increase patient harm

- Nearly 32 million people in the US report a penicillin allergy
- Over 95% of patients who report a penicillin allergy and undergo penicillin allergy testing do not have an allergy
- Reported penicillin allergies increase use of alternative antibiotics (clindamycin, fluoroquinolones, vancomycin), exposing patients to harm
    - ↑ rates of surgical site infections (OR=1.51; 95% CI 1.02-2.22)
    - ↑ postprocedural acute kidney injury (7.5% vs. 0.6%, p >0
    - ↑ *C. difficile* infection (HR=1.26; 95% CI 1.12 - 1.40)

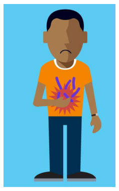

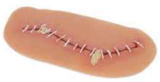

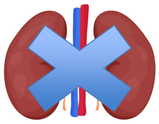

Bhathal S et al. Surgery. 2022 Dec;172(6):1598-1603
Macy E J Allergy Clin Immunol. 2014; 133:790–6
Blumenthal KG et al. Clin Infect Dis. 2018 Jan 18;66(3):329-336

2

2

Misclassified penicillin allergies contribute to the global health threat of antibiotic resistance

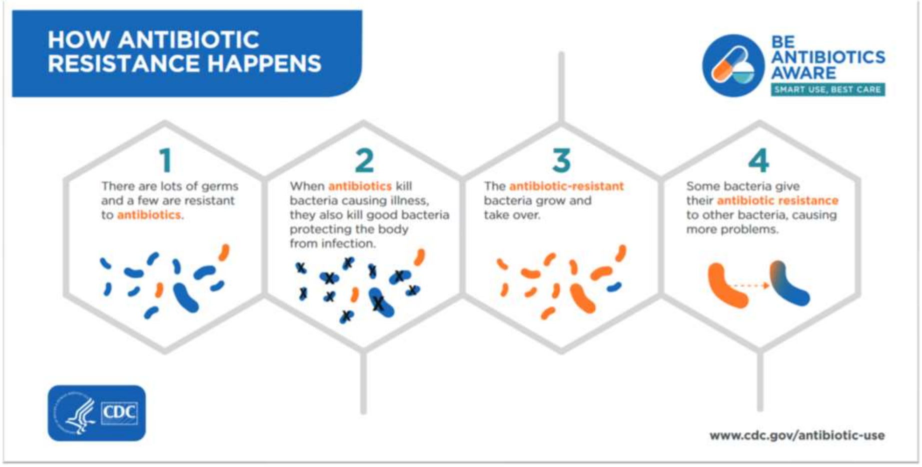

3

3

Evaluation & management of penicillin allergies

Endorsed by:

- American Academy of Allergy, Asthma, and Immunology (AAAAI)
- Infectious Diseases Society of America (IDSA)
- Society for Healthcare Epidemiology of America (SHEA)

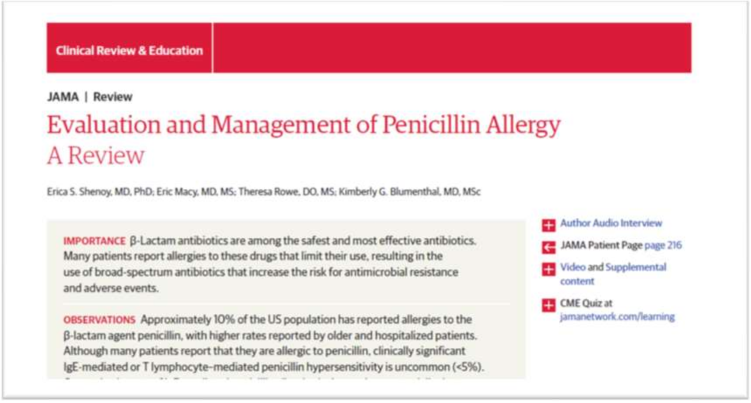

Clinical Review & Education

JAMA | Review

Evaluation and Management of Penicillin Allergy
A Review

Erica S. Shenoy, MD, PhD; Eric Macy, MD, MS; Theresa Rowe, DO, MS; Kimberly G. Blumenthal, MD, MSc

IMPORTANCE β-Lactam antibiotics are among the safest and most effective antibiotics. Many patients report allergies to these drugs that limit their use, resulting in the use of broad-spectrum antibiotics that increase the risk for antimicrobial resistance and adverse events.

OBSERVATIONS Approximately 10% of the US population has reported allergies to the β-lactam agent penicillin, with higher rates reported by older and hospitalized patients. Although many patients report that they are allergic to penicillin, clinically significant IgE-mediated or T lymphocyte–mediated penicillin hypersensitivity is uncommon (<5%).

Author Audio Interview
JAMA Patient Page page 216
Video and Supplemental content
CME Quiz at jamanetwork.com/learning

Shenoy, Erica S., et al. "Evaluation and management of penicillin allergy: a review." *JAMA* 321.2 (2019): 188-199.

4

4

Penicillin allergy toolkits included in review

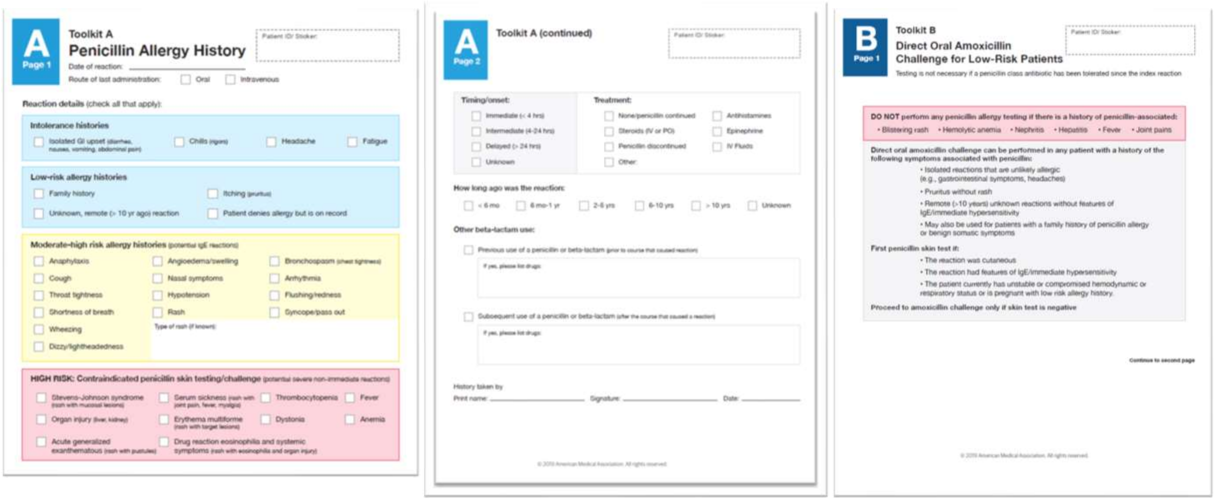
Toolkit A
Penicillin Allergy History
Patient ID/ Sticker:
Date of reaction:
Route of last administration: ☐ Oral ☐ Intravenous

Reaction details (check all that apply):

Intolerance histories
☐ Isolated GI upset (diarrhea, nausea, vomiting, abdominal pain) ☐ Chills (rigors) ☐ Headache ☐ Fatigue

Low-risk allergy histories
☐ Family history ☐ Itching (pruritus)
☐ Unknown, remote (> 10 yr ago) reaction ☐ Patient denies allergy but is on record

Moderate-high risk allergy histories (potential IgE reactions)
☐ Anaphylaxis ☐ Angioedema/swelling ☐ Bronchospasm (chest tightness)
☐ Cough ☐ Nasal symptoms ☐ Arrhythmia
☐ Throat tightness ☐ Hypotension ☐ Flushing/redness
☐ Shortness of breath ☐ Rash ☐ Syncope/pass out
☐ Wheezing Type of rash (if known):
☐ Dizzy/lightheadedness

HIGH RISK: Contraindicated penicillin skin testing/challenge (potential severe non-immediate reactions)
☐ Stevens-Johnson syndrome (rash with mucosal lesions) ☐ Serum sickness (rash with joint pain, fever, myalgia) ☐ Thrombocytopenia ☐ Fever
☐ Organ injury (liver, kidney) ☐ Erythema multiforme (rash with target lesions) ☐ Dystonia ☐ Anemia
☐ Acute generalized exanthematous (rash with pustules) ☐ Drug reaction eosinophilia and systemic symptoms (rash with eosinophilia and organ injury)

Toolkit A (continued)
Patient ID/ Sticker:

Timing/onset:
☐ Immediate (< 4 hrs) ☐ Intermediate (4-24 hrs) ☐ Delayed (> 24 hrs) ☐ Unknown

Treatment:
☐ None/penicillin continued ☐ Antihistamines ☐ Steroids (IV or PO) ☐ Epinephrine ☐ Penicillin discontinued ☐ IV Fluids ☐ Other:

How long ago was the reaction:
☐ < 6 mo ☐ 6 mo-1 yr ☐ 2-5 yrs ☐ 6-10 yrs ☐ > 10 yrs ☐ Unknown

Other beta-lactam use:
☐ Previous use of a penicillin or beta-lactam (prior to course that caused reaction)
If yes, please list drugs:
☐ Subsequent use of a penicillin or beta-lactam (after the course that caused a reaction)
If yes, please list drugs:

History taken by
Print name: Signature: Date:

Toolkit B
Direct Oral Amoxicillin Challenge for Low-Risk Patients
Patient ID/ Sticker:
Testing is not necessary if a penicillin class antibiotic has been tolerated since the index reaction

DO NOT perform any penicillin allergy testing if there is a history of penicillin-associated:
• Blistering rash • Hemolytic anemia • Nephritis • Hepatitis • Fever • Joint pains

Direct oral amoxicillin challenge can be performed in any patient with a history of the following symptoms associated with penicillin:
• Isolated reactions that are unlikely allergic (e.g., gastrointestinal symptoms, headaches)
• Pruritus without rash
• Remote (>10 years) unknown reactions without features of IgE/immediate hypersensitivity
• May also be used for patients with a family history of penicillin allergy or benign somatic symptoms

First penicillin skin test if:
• The reaction was cutaneous
• The reaction had features of IgE/immediate hypersensitivity
• The patient currently has unstable or compromised hemodynamic or respiratory status or is pregnant with low risk allergy history.

Proceed to amoxicillin challenge only if skin test is negative

Continue to second page

Shenoy, Erica S., et al. "Evaluation and management of penicillin allergy: a review." *JAMA* 321.2 (2019): 188-199.

Critical missing piece

- One-fifth of documented penicillin allergies have an unknown/undocumented reaction, precluding risk stratification

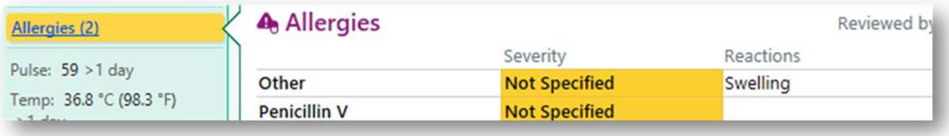

Albin, Stephanie, and Shradha Agarwal. "Prevalence and characteristics of reported penicillin allergy in an urban outpatient adult population." *Allergy and asthma proceedings*. Vol. 35. No. 6.

Staicu, Mary L., et al. "Penicillin allergy delabeling: a multidisciplinary opportunity." *The Journal of Allergy and Clinical Immunology: In Practice* 8.9 (2020): 2858-2868.

Nurses play a critical role in this space.

IF WE DON'T FIX INACCURATE REPORTS OF PENICILLIN ALLERGY, WE PERPETUATE ANTIBIOTIC RESISTANCE AND MAY CONTRIBUTE TO OTHER WORSE PATIENT OUTCOMES.

7

7

The CDC encourages nurses to improve the evaluation of reported penicillin allergies

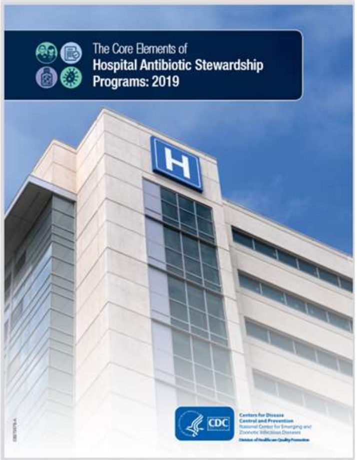

Nurses: There is growing recognition of the importance of engaging nurses in hospital stewardship efforts[27-29]. Nurses can play an especially important role in:

- Optimizing testing, or diagnostic stewardship. For example, nurses can inform decisions about whether or not a patient has symptoms that might justify a urine culture.
- Assuring that cultures are performed correctly before starting antibiotics.
- Prompting discussions of antibiotic treatment, indication, and duration.
- Improving the evaluation of penicillin allergies.

8

8

Examples of penicillin antibiotics include

- Amoxicillin
- Ampicillin
- Augmentin (amoxicillin-clavulanate)
- Nafcillin
- Oxacillin
- Penicillin
- Unasyn (ampicillin-sulbactam)
- Zosyn (piperacillin-tazobactam)

9

9

Misclassified penicillin allergies
Nurses are very familiar with this problem

"It's really tricky for the nurse because…you say, 'What are your allergies?' and they tell you. And then you say, 'What's your reaction when you have them?' and they tell you. And you know that this is really a sensitivity, and this patient is not allergic to [Penicillin]."

– Nurse

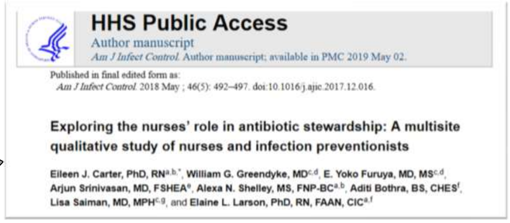
HHS Public Access

Author manuscript

Am J Infect Control. Author manuscript; available in PMC 2019 May 02.

Published in final edited form as:

Am J Infect Control. 2018 May ; 46(5): 492–497. doi:10.1016/j.ajic.2017.12.016.

Exploring the nurses' role in antibiotic stewardship: A multisite qualitative study of nurses and infection preventionists

Eileen J. Carter, PhD, RN[a,b,*], William G. Greendyke, MD[c,d], E. Yoko Furuya, MD, MS[c,d], Arjun Srinivasan, MD, FSHEA[e], Alexa N. Shelley, MS, FNP-BC[a,b], Aditi Bothra, BS, CHES[f], Lisa Saiman, MD, MPH[c,g], and Elaine L. Larson, PhD, RN, FAAN, CIC[a,f]

10

10

Previous work: what nurses perceived as important and feasible

Feasible and Important

- Nurses comprehensively assess and document reported penicillin allergies
- Nurses alert prescribers of patients who have low-risk symptoms of reported penicillin allergies

Barriers to Implementation

- What questions should nurses ask when assessing reported penicillin allergies?
- What are low-risk symptoms of reported penicillin allergy?

Carter E.J., et al. "Exploring the nurses' role in antibiotic stewardship." *Am J Infect Control* 46.5 (2018): 492-497.

11

11

Current State

The documentation of penicillin allergies is often incomplete and lacks critical information needed for risk stratification.

Perioperative nurses routinely assess the allergy histories of patients undergoing surgery and document this information in electronic medical records. This information is reviewed by OR nurses.

Surgeons use this information to guide selection of antibiotic prophylaxis.

12

12

To Improve the Evaluation of Penicillin Allergies, Nurses are asked to:

1. Document reported penicillin allergies using STORY
2. Notify providers of patients with low-risk symptoms of reported penicillin allergy
3. Enter an Antimicrobial Stewardship note regarding patients reporting a penicillin allergy with low-risk allergy symptoms

13

13

Tools that are Responsive to Nurse Needs

- We carefully designed tools to facilitate nurses' evaluation of reported penicillin allergies
- These tools were designed with YOU in mind
  - Address concerns identified in previous work
  - Add value
  - Minimize redundancy & unnecessary effort
  - Fit into current workflow

14

14

To Improve the Evaluation of Penicillin Allergies, Nurses are asked to:

1. Document reported penicillin allergies using STORY
2. Notify providers of patients with low-risk symptoms of reported penicillin allergy
3. Enter an Antimicrobial Stewardship note regarding patients reporting a penicillin allergy with low-risk allergy symptoms

15

15

"**STORY**" to facilitate nurses' thorough evaluation of reported penicillin allergies

STORY mnemonic acronym

**S**ymptoms	Describe the *symptoms* of the patient's reaction to penicillin.
**T**iming	Indicate how much *time* (in years) has passed since reaction.
**O**nset	Specify the *onset* of the reaction (e.g., started immediately after taking penicillin, within 24 hours of taking penicillin, etc.).
**R**esolution	Describe how symptoms *resolved* or went away.
**Y**et again	Specify whether the patient has received penicillin *yet again* since the reaction and it so, how penicillin was tolerated.

Pocket Card

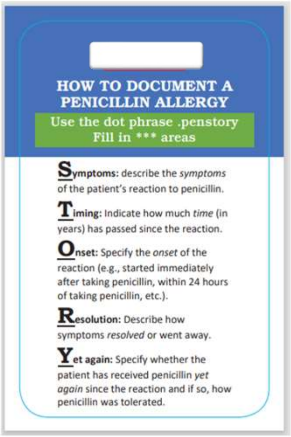

16

16

Let’s walk-through **.penstory** (1/2)

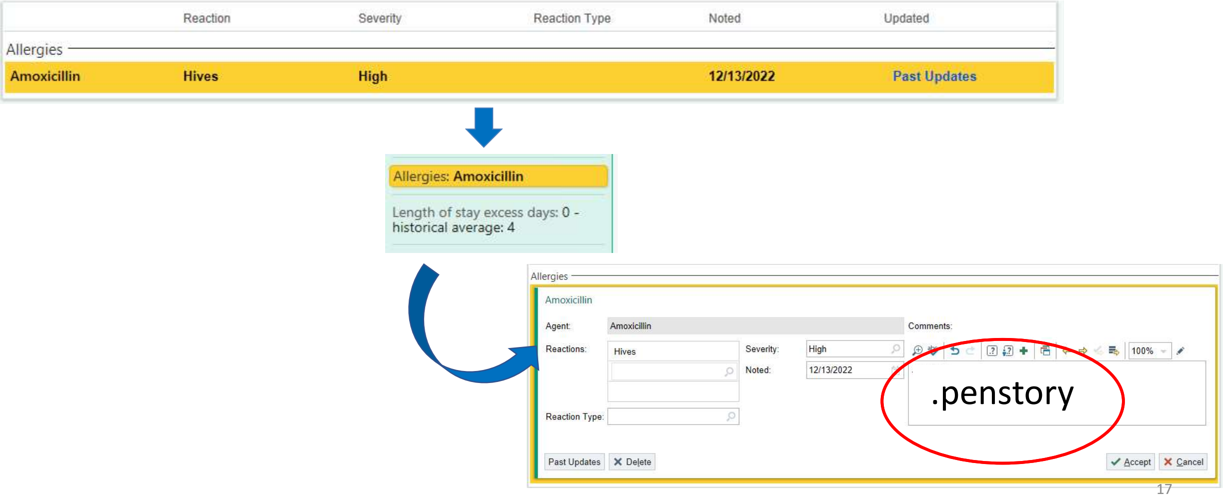

17

Let’s walk through **.penstory** (2/2)

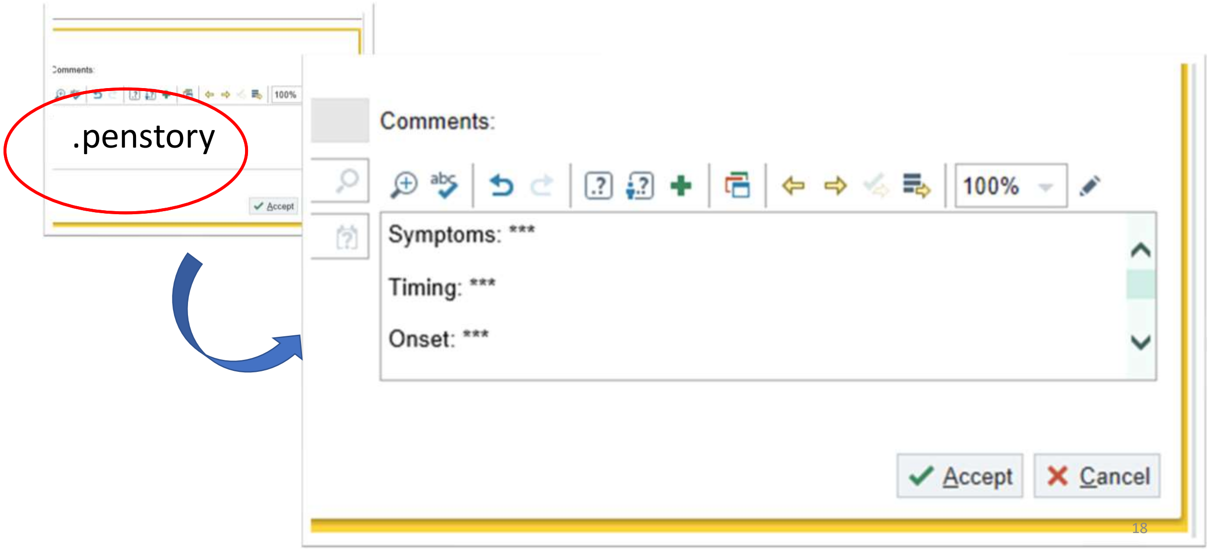

18

To Improve the Evaluation of Penicillin Allergies, Nurses are asked to:

1. Document reported penicillin allergies using STORY
2. Notify providers of patients with low-risk symptoms of reported penicillin allergy
3. Enter an Antimicrobial Stewardship note regarding patients reporting a penicillin allergy with low-risk allergy symptoms

19

19

Pocket Card Details Low-Risk Allergy Symptoms

- Orally notify providers of patients with low-risk symptoms of reported penicillin allergy

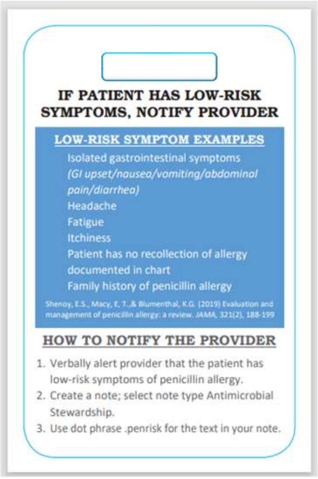

20

20

To Improve the Evaluation of Penicillin Allergies, Nurses are asked to:

1. Document reported penicillin allergies using STORY
2. Notify providers of patients with low-risk symptoms of reported penicillin allergy
3. Enter an Antimicrobial Stewardship note regarding patients reporting a penicillin allergy with low-risk allergy symptoms

21

21

Pocket card details how to enter antimicrobial stewardship note

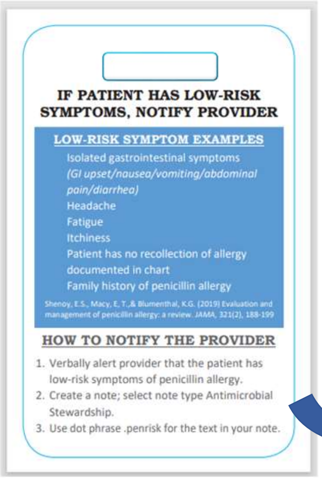

- Create a Note
- Select note Type Antimicrobial Stewardship
- Use dot phrase .penrisk

22

22

Let’s walk through **.penrisk** (1/2)

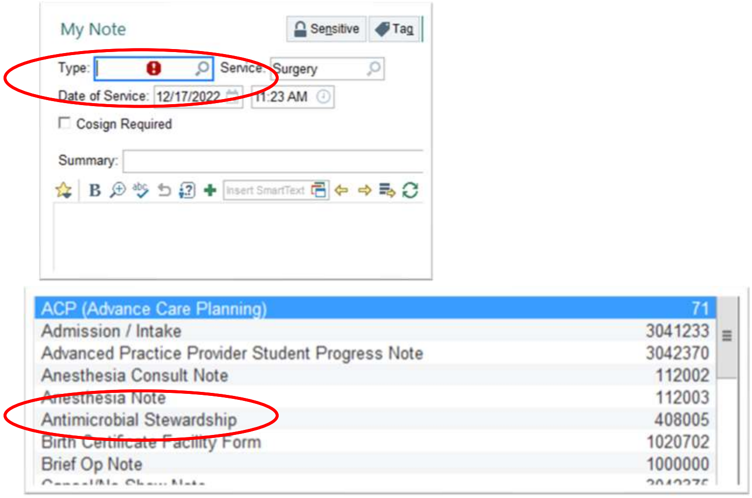

.penrisk

23

23

Let’s walk through **.penrisk** (2/2)

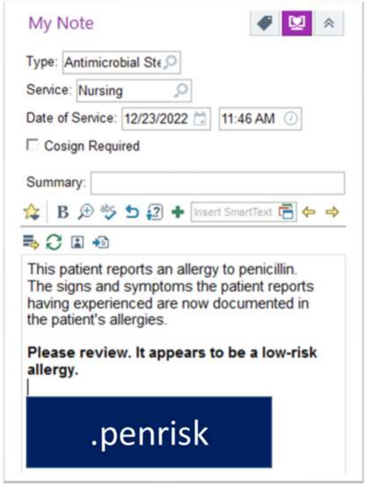

24

24

RECAP
Nurses are asked to:

1. Document reported penicillin allergies using STORY

   .penstory

2. Notify providers of patients with low-risk symptoms of reported penicillin allergy

3. Enter an Antimicrobial Stewardship note regarding patients reporting a penicillin allergy with low-risk allergy symptoms

   .penrisk

25

Initial prescriber feedback

- ✓ Nurses play an important role in assessing penicillin allergies.
- ✓ STORY is clinically relevant.
- ✓ STORY will contribute to medical history taking.
- ✓ STORY seems useful in the clinical management of patients.

26

Next steps

- Compare the completeness of penicillin allergy documentation pre- and post-introduction of STORY
- Evaluate nurses' use of the antibiotic stewardship note post-introduction of the STORY acronym
- Evaluate the completeness of penicillin allergy history documentation on the selection of antibiotic prophylaxis
- Expand STORY

Funding: The Research Foundation of the Competency and Credentialing Institute

27

27

Are you interested in learning more?

Please contact:
David Banach, MD
dbanach@uchc.edu

Eileen Carter, PhD, RN
eileen.carter@uconn.edu

THANK YOU

28

28

References

- American Nurses Association & Centers for Disease Control and Prevention. Redefining the Antibiotic Stewardship Team: Recommendations from the American Nurses Association/Centers for Disease Control and Prevention Workgroup on the Role of Registered Nurses in Hospital Antibiotic Stewardship Practices. Silver Springs, MD: American Nurses Association; 2017.
- Blumenthal KG, Ryan EE, Li Y, Lee H, Kuhlen JL, Shenoy ES. The Impact of a Reported Penicillin Allergy on Surgical Site Infection Risk. Clin Infect Dis 2018;66:329-36.
- Carter EJ, Greendyke WG, Furuya EY, et al. Exploring the nurses' role in antibiotic stewardship: A multisite qualitative study of nurses and infection preventionists. Am J Infect Control 2018;46:492-7.
- Centers for Disease Control and Prevention. Antibiotic Resistance Threats in the United States: U.S. Department of Health and Human Services,; 2019.
- Inglis JM, Caughey GE, Smith W, Shakib S. Documentation of Penicillin Adverse Drug Reactions in Electronic Health Records: Inconsistent Use of Allergy and Intolerance Labels. Internal medicine journal 2017.
- Shenoy ES, Macy E, Rowe T, Blumenthal KG. Evaluation and Management of Penicillin Allergy: A Review. Jama 2019;321:188-99
- Vyles D, Chiu A, Simpson P, Nimmer M, Adams J, Brousseau DC. Parent-Reported Penicillin Allergy Symptoms in the Pediatric Emergency Department. Acad Pediatr 2017;17:251-5.
- Wilhelm NB, Bonsall TJ, Miller CL. The Effect of Beta-lactam Allergy Status on the Rate of Surgical Site Infections: A Retrospective Cohort Study. Ann Surg 2022;275:208-12.

Appendix C. Rationale for Implementation Strategy

Implementation Strategy	Rationale for Implementation Strategy
Build a coalition<br><br>Purposely reexamine the implementation	We built a coalition and purposely reexamined the implementation effort because nurses' successful adoption of evidence-based interventions have been accompanied by the ongoing engagement of interdisciplinary stakeholders, in which implementation strategies are purposely, developed, examined, and adapted as necessary.[1,2]
Inform local opinion leaders	We informed nursing leadership and members of the antimicrobial stewardship committee of the initiative to influence a workplace culture that esteems nurses' partnership in antibiotic stewardship.[3]
Conducted educational meetings with perioperative nurses and surgical providers.	We conducted educational meetings with perioperative nurses to address barriers and amplify facilitators to nurses' evaluation of penicillin allergies.[4,5] Barriers include a lack of awareness of the harms of documented penicillin allergy, a lack of awareness of penicillin allergy assessment fields, and a lack of awareness of penicillin reaction symptoms that suggest an intolerance or low-risk penicillin allergy.[4,5] Facilitators include nurses' desire to improve patient care and outcomes, nurses' routine assessment of patient allergy histories, and nurses' willingness to take a detailed penicillin allergy assessment.[4,5]<br><br>We conducted educational meetings with surgical providers prior to the rollout of the initiative to ensure providers' support of the initiative as provider push-back has hindered nurses' active engagement in the prevention of hospital-acquired infections.[6,7]
Developed and distributed educational materials (i.e., pocket cards)	We developed and distributed pocket cards to perioperative nurses to reinforce the material provided during the educational meeting and to serve as a quick reference for nurses at the point of care. Pocket cards are frequently used in translational research initiatives among nurses.[8-10]
Change record systems	We implemented dot phrases in the electronic medical record to minimize documentation burden associated with the initiative, as documentation burden is already high among nurses and may impact burnout.[11]
Obtain and use feedback	We shared positive feedback concerning nurses' participation in the initiative with perioperative nurses as nurses are motivated by the perceived meaningfulness and impact of their work.[12]

REFERENCES

1. Dykes PC, Burns Z, Adelman J, et al. Evaluation of a Patient-Centered Fall-Prevention Tool Kit to Reduce Falls and Injuries: A Nonrandomized Controlled Trial. *JAMA Netw Open*. 2020;3(11):e2025889.
2. Carter EJ, Pallin DJ, Mandel L, Sinnette C, Schuur JD. A Qualitative Study of Factors Facilitating Clinical Nurse Engagement in Emergency Department Catheter-Associated Urinary Tract Infection Prevention. *J Nurs Adm*. 2016;46(10):495-500.
3. Monsees EA, Tamma PD, Cosgrove SE, Miller MA, Fabre V. Integrating bedside nurses into antibiotic stewardship: A practical approach. *Infection control and hospital epidemiology*. 2019;40(5):579-584.
4. Carter EJ, Greendyke WG, Furuya EY, et al. Exploring the nurses' role in antibiotic stewardship: A multisite qualitative study of nurses and infection preventionists. *American journal of infection control*. 2018;46(5):492-497.
5. Monsees E, Popejoy L, Jackson MA, Lee B, Goldman J. Integrating staff nurses in antibiotic stewardship: Opportunities and barriers. *American journal of infection control*. 2018;46(7):737-742.
6. DePuccio MJ, Gaughan AA, Sova LN, et al. An Examination of the Barriers to and Facilitators of Implementing Nurse-Driven Protocols to Remove Indwelling Urinary Catheters in Acute Care Hospitals. *Jt Comm J Qual Patient Saf*. 2020;46(12):691-698.
7. McAlearney AS, Hefner JL. Facilitating central line-associated bloodstream infection prevention: a qualitative study comparing perspectives of infection control professionals and frontline staff. *American journal of infection control*. 2014;42(10 Suppl):S216-222.
8. Rutten JJS, Smalbrugge M, van Buul LW, et al. A Process Evaluation of an Antibiotic Stewardship Intervention for Urinary Tract Infections in Nursing Homes. *Journal of the American Medical Directors Association*. 2024;25(1):146-154.e149.
9. Pun BT, Gordon SM, Peterson JF, et al. Large-scale implementation of sedation and delirium monitoring in the intensive care unit: a report from two medical centers. *Crit Care Med*. 2005;33(6):1199-1205.
10. Schmidt T, Kocher DR, Mahendran P, Denecke K. Dynamic Pocket Card for Implementing ISBAR in Shift Handover Communication. *Stud Health Technol Inform*. 2019;267:224-229.
11. Gesner E, Dykes PC, Zhang L, Gazarian P. Documentation Burden in Nursing and Its Role in Clinician Burnout Syndrome. *Appl Clin Inform*. 2022;13(5):983-990.
12. Moody RC, Pesut DJ. The motivation to care: application and extension of motivation theory to professional nursing work. *J Health Organ Manag*. 2006;20(1):15-48.

Appendix D. Standards for Reporting Implementation Studies: the StaRI checklist

The StaRI standard should be referenced as: Pinnock H, Barwick M, Carpenter C, Eldridge S, Grandes G, Griffiths CJ, Rycroft-Malone J, Meissner P, Murray E, Patel A, Sheikh A, Taylor SJC for the StaRI Group. Standards for Reporting Implementation Studies (StaRI) statement. *BMJ* 2017;356:i6795

The detailed Explanation and Elaboration document, which provides the rationale and exemplar text for all these items is: Pinnock H, Barwick M, Carpenter C, Eldridge S, Grandes G, Griffiths C, Rycroft-Malone J, Meissner P, Murray E, Patel A, Sheikh A, Taylor S, for the StaRI group. Standards for Reporting Implementation Studies (StaRI). Explanation and Elaboration document. *BMJ Open* 2017 2017;7:e013318

Notes: A key concept of the StaRI standards is the dual strands of describing, on the one hand, the implementation strategy and, on the other, the clinical, healthcare, or public health intervention that is being implemented. These strands are represented as two columns in the checklist.

The primary focus of implementation science is the implementation strategy (column 1) and the expectation is that this will always be completed.	The evidence about the impact of the intervention on the targeted population should always be considered (column 2) and either health outcomes reported or robust evidence cited to support a known beneficial effect of the intervention on the health of individuals or populations.

The StaRI standard refers to the broad range of study designs employed in implementation science. Authors should refer to other reporting standards for advice on reporting specific methodological features. Conversely, whilst all items are worthy of consideration, not all items will be applicable to, or feasible within every study.

Checklist item		Reported on page #	Implementation Strategy	Reported on page #	Intervention
		**5**	"Implementation strategy" refers to how the intervention was implemented	**5**	"Intervention" refers to the healthcare or public health intervention that is being implemented.
**Title and abstract**					
Title	**1**	1, 6	Identification as an implementation study, and description of the methodology in the title and/or keywords		
Abstract	**2**	abstract	Identification as an implementation study, including a description of the implementation strategy to be tested, the evidence-based intervention being implemented, and defining the key implementation and health outcomes.		
**Introduction**					
Introduction	**3**	4	Description of the problem, challenge or deficiency in healthcare or public health that the intervention being implemented aims to address.		
Rationale	**4**	Appendix B	The scientific background and rationale for the implementation strategy (including any underpinning	5	The scientific background and rationale for the intervention being implemented (including evidence

			theory/framework/model, how it is expected to achieve its effects and any pilot work).		about its effectiveness and how it is expected to achieve its effects).
Aims and objectives	**5**	6	The aims of the study, differentiating between implementation objectives and any intervention objectives.		
**Methods: description**					
Design	**6**	6	The design and key features of the evaluation, (cross referencing to any appropriate methodology reporting standards) and any changes to study protocol, with reasons		
Context	**7**	6	The context in which the intervention was implemented. (Consider social, economic, policy, healthcare, organisational barriers and facilitators that might influence implementation elsewhere).		
Targeted 'sites'	**8**	5	The characteristics of the targeted 'site(s)' (e.g locations/personnel/resources etc.) for implementation and any eligibility criteria.	6	The population targeted by the intervention and any eligibility criteria.
Description	**9**	5, Table 1	A description of the implementation strategy	Appendix A	A description of the intervention
Sub-groups	**10**	8	Any sub-groups recruited for additional research tasks, and/or nested studies are described		
**Methods: evaluation**					
Outcomes	**11**	7	Defined pre-specified primary and other outcome(s) of the implementation strategy, and how they were assessed. Document any pre-determined targets	NA	Defined pre-specified primary and other outcome(s) of the intervention (if assessed), and how they were assessed. Document any pre-determined targets
Process evaluation	**12**	NA	Process evaluation objectives and outcomes related to the mechanism by which the strategy is expected to work		
Economic evaluation	**13**	NA	Methods for resource use, costs, economic outcomes and analysis for the implementation strategy	NA	Methods for resource use, costs, economic outcomes and analysis for the intervention
Sample size	**14**	7, 15	Rationale for sample sizes (including sample size calculations, budgetary constraints, practical considerations, data saturation, as appropriate)		
Analysis	**15**	7, 8	Methods of analysis (with reasons for that choice)		
Sub-group analyses	**16**	Table 3	Any a priori sub-group analyses (e.g. between different sites in a multicentre study, different clinical or demographic populations), and sub-groups recruited to specific nested research tasks		

**Results**					
Characteristics	**17**	9	Proportion recruited and characteristics of the recipient population for the implementation strategy	NA	Proportion recruited and characteristics (if appropriate) of the recipient population for the intervention
Outcomes	**18**	Table 2, Table 3	Primary and other outcome(s) of the implementation strategy	NA	Primary and other outcome(s) of the Intervention (if assessed)
Process outcomes	**19**	11	Process data related to the implementation strategy mapped to the mechanism by which the strategy is expected to work		
Economic evaluation	**20**	15	Resource use, costs, economic outcomes and analysis for the implementation strategy	NA	Resource use, costs, economic outcomes and analysis for the intervention
Sub-group analyses	**21**	Table 3	Representativeness and outcomes of subgroups including those recruited to specific research tasks		
Fidelity/ adaptation	**22**	11	Fidelity to implementation strategy as planned and adaptation to suit context and preferences	NA	Fidelity to delivering the core components of intervention (where measured)
Contextual changes	**23**	NA	Contextual changes (if any) which may have affected outcomes		
Harms	**24**	11	All important harms or unintended effects in each group		
**Discussion**					
Structured discussion	**25**	14, 15	Summary of findings, strengths and limitations, comparisons with other studies, conclusions and implications		
Implications	**26**	14	Discussion of policy, practice and/or research implications of the implementation strategy (specifically including scalability)	NA	Discussion of policy, practice and/or research implications of the intervention (specifically including sustainability)
**General**					
Statements	**27**	9	Include statement(s) on regulatory approvals (including, as appropriate, ethical approval, confidential use of routine data, governance approval), trial/study registration (availability of protocol), funding and conflicts of interest		
